# Plasma clearance, biodistribution and therapeutic properties of mitoxantrone encapsulated in conventional and sterically stabilized liposomes after intravenous administration in BDF1 mice.

**DOI:** 10.1038/bjc.1997.28

**Published:** 1997

**Authors:** C. W. Chang, L. Barber, C. Ouyang, D. Masin, M. B. Bally, T. D. Madden

**Affiliations:** University of British Columbia, Department of Pharmacology and Therapeutics, Vancouver, Canada.

## Abstract

Mitoxantrone can be efficiently loaded into large unilamellar vesicles using a transmembrane pH gradient. Release studies indicate that these drug-loaded carriers are highly stable and even after dissipation of the residual pH gradient retain more than 85% of encapsulated mitoxantrone following dialysis at 37 degrees C for 5 days. In murine studies we have compared the plasma clearance and biodistribution of both mitoxantrone and liposomal lipid following intravenous administration of free drug or mitoxantrone encapsulated in either conventional or sterically stabilized liposomes. In contrast to the rapid blood clearance observed for free mitoxantrone, both liposomal systems provided extended circulation lifetimes, with over 90% of the drug present 1 h after administration and 15-30% remaining at 24 h. In agreement with previous reports, longer plasma half-lives were observed for sterically stabilized liposomes than for conventional systems. In addition, a strong correlation between drug and carrier biodistribution was seen, with uptake occurring mainly in the liver and spleen and paralleling plasma clearance. This would suggest that tissue disposition reflects that of drug-loaded liposomes rather than the individual components. Liposomal encapsulation also significantly reduced mitoxantrone toxicity, allowing administration of higher, more efficacious drug doses. In a murine L1210 tumour model, for example, no long-term survivors were seen in animal groups treated with free drug, whereas at the maximum therapeutic dose of liposomal mitoxantrone survival rates of 40% were observed.


*British Journal of Cancer* (1997) **75**(2), 169–177
© 1997 Cancer Research Campaign

### Plasma clearance, biodistribution and therapeutic properties of mitoxantrone encapsulated in conventional and sterically stabilized liposomes after intravenous administration in BDF1 mice

**CW Chang[1], L Barber[1], C Ouyang[1], D Masin[2], MB Bally[2] and TD Madden[1]**

[1]University of British Columbia, Department of Pharmacology and Therapeutics, Vancouver, British Columbia, Canada V6T 1Z3; [2]Division of Medical Oncology, British Columbia Cancer Agency, 600 West 10th Avenue, Vancouver, British Columbia, Canada V5Z 4E6

**Summary** Mitoxantrone can be efficiently loaded into large unilamellar vesicles using a transmembrane pH gradient. Release studies indicate that these drug-loaded carriers are highly stable and even after dissipation of the residual pH gradient retain more than 85% of encapsulated mitoxantrone following dialysis at 37°C for 5 days. In murine studies we have compared the plasma clearance and biodistribution of both mitoxantrone and liposomal lipid following intravenous administration of free drug or mitoxantrone encapsulated in either conventional or sterically stabilized liposomes. In contrast to the rapid blood clearance observed for free mitoxantrone, both liposomal systems provided extended circulation lifetimes, with over 90% of the drug present 1 h after administration and 15–30% remaining at 24 h. In agreement with previous reports, longer plasma half-lives were observed for sterically stabilized liposomes than for conventional systems. In addition, a strong correlation between drug and carrier biodistribution was seen, with uptake occurring mainly in the liver and spleen and paralleling plasma clearance. This would suggest that tissue disposition reflects that of drug-loaded liposomes rather than the individual components. Liposomal encapsulation also significantly reduced mitoxantrone toxicity, allowing administration of higher, more efficacious drug doses. In a murine L1210 tumour model, for example, no long-term survivors were seen in animal groups treated with free drug, whereas at the maximum therapeutic dose of liposomal mitoxantrone survival rates of 40% were observed.

**Keywords**: mitoxantrone; liposome; anti-tumour efficacy; polyethylene glycol–lipid; biodistribution

Liposomes have been widely employed as carrier systems for anticancer drugs. These microscopic lipid systems tend to accumulate within organs of the reticuloendothelial system (RES) and also at disease sites, including tumours (Morgan, et al, 1985; Ogihara et al, 1986; Presant et al, 1986; Williams et al, 1986). As a result, they offer the potential to modify both the pharmacokinetics and biodistribution of entrapped drugs. In the case of doxorubicin, this results in a significant reduction in drug levels within the heart and a corresponding decrease in cardiotoxicity (Rahman et al, 1980; Gabizon et al, 1982; Olson et al, 1982; Mayer et al, 1989). Similar benefits have been reported for other anthracycline antineoplastics, including daunorubicin and epirubicin (Forssen, 1988; Gabizon, 1992). Further, in the case of the antimitotic agent vincristine, liposomal encapsulation has been shown to both reduce drug toxicity and enhance efficacy (Mayer et al, 1990a; Boman et al, 1994). This improvement in antineoplastic activity is believed to result from sustained drug release following accumulation of the liposomal carrier at the tumour site (Boman et al, 1994). As a cell cycle-specific agent, vincristine cytotoxicity is highly dependent on exposure time.

As noted above, following intravenous administration, conventional liposomes tend to be cleared from the circulation by phagocytic cells within the liver and spleen. Liposome recognition and subsequent uptake is believed to be triggered by protein binding to the vesicle surface (Chonn, et al, 1992; Funalo et al, 1992). This protein binding can be inhibited, however, by introducing into the liposomal membrane lipid derivatives possessing long, hydrophilic poly(ethylene glycol) chains. These sterically stabilized liposomes exhibit much longer blood circulation times than equivalent conventional liposomes (Klibanov et al, 1990; Allen et al, 1991) and when used as drug carriers can enhance delivery to tumour sites, resulting in improved anti-cancer efficacy (Papajopoulos et al, 1991; Huang et al, 1992; Vaage et al, 1992).

Mitoxantrone is an anthracenedione derivative that shows good antineoplastic activity against breast cancer, leukaemia and lymphoma and this drug appears to exhibit less cardiotoxicity than doxorubicin (Smith, 1983; Shenkenberg and Von Hoff, 1986). A liposomal formulation of this agent has been reported to show lower toxicity and, in some instances, greater efficacy than the free drug (Schwendener et al, 1991; Pestalozzi et al, 1992). The liposomal formulation employed in these previous studies, however, consisted of an electrostatic complex between the cationic drug and liposomes containing acidic phospholipids, and relatively rapid plasma clearance of the drug was observed following intravenous administration (Schwendener et al, 1991). In the present study, therefore, we have developed systems in which mitoxantrone is encapsulated within the liposome aqueous interior, employing a transmembrane pH gradient to drive drug uptake. We then compared the pharmacokinetics and therapeutic properties of conventional liposome carriers and sterically stabilized liposomes.

*Received 28 November 1995*
*Revised 31 May 1996*
*Accepted 21 August 1996*

*Correspondence to:* TD Madden

### MATERIALS AND METHODS

Mitoxantrone hydrochloride (Novantrone) was obtained from Cyanamid Canada (Montreal, Quebec) in saline solution (2.0 mg $ml^{-1}$). Distearoylphosphatidylcholine (DSPC) and poly(ethylene glycol)-dipalmitoylphosphatidylethanolamine ($DPPE-PEG_{2000}$) were purchased from Avanti Polar Lipids (Birmingham, AL, USA). Cholesterol (standard for chromatography) was obtained from Sigma Chemicals (St Louis, MO, USA). [$^3$H]Cholesterol hexadecyl ether and [$^3$H]dipalmitoylphosphatidylcholine (DPPC) were supplied by NEN (DuPont Canada, Mississauga, Ontario) and [$^{14}$C]mitoxantrone (10 μCi 100 $\mu l^{-1}$) was a generous gift from Lederle. Packard Ultima Gold liquid scintillation cocktail was obtained from Packard Instrument Company (Meriden, CT, USA). Nigericin (90–95% pure) was obtained from Sigma and prepared in ethanol. BDFI and CD1 mice (8–9 weeks old, 18.0–23.0 g) were purchased from Charles River and were initially housed in microisolation cages (five mice per cage) during a 1-week quarantine period. Mice were then moved to conventional Nalgene cages and maintained on wood shavings. Animal rooms followed a 12-h day/night cycle with mean temperature 22°C and mean humidity 35%. Mice were fed standard, certified commercial mouse food (LabDiet, The Richmond Standard) and provided with municipal tap water, ad libitum. Normal (pooled) mouse serum (CL8000) was produced by Cedar Lane Laboratories and stored at −10°C or below. All other reagents, salts, buffers and organic solvents were of analytical reagent grade or better.

### Preparation of large unilamellar vesicles (LUVs)

The lipid mixtures DSPC/cholesterol (Chol) (55:45 molar ratio) and DSPC/Chol/$DPPE-PEG_{2000}$ (50:45:5 molar ratio) were dissolved in benzene – methanol (95:5, v/v), rapidly frozen in liquid nitrogen and then lyophilized at ≤60 mT for a minimum of 5 h (Virtis lyophilizer with liquid nitrogen cold trap). During lyophilization, the lipid mixture was protected from light.

Multilamellar vesicles (MLVs) were prepared by hydrating the dry lipid mixture in 300 mM citrate pH 4.0 at the desired phospholipid concentration. Following incubation at 60°C for 5 min with occasional vortexing, the sample was transferred to a 5 ml cryovial and taken through five freeze–thaw cycles employing liquid nitrogen and incubation at 60°C (Mayer et al, 1986*a*). Large unilamellar vesicles were prepared from these frozen and thawed MLVs by extruding the sample through three stacked 100-nm-pore size polycarbonate filters (20 times) at 65°C using an Extruder (Lipex Biomembranes, Vancouver, BC, Canada) (Hope et al, 1985). A transmembrane proton gradient was established by passing an aliquot (1.2 ml) of the LUV suspension down a Sephadex G-50 (medium) column (1.0 cm × 15.0 cm) equilibrated with degassed 150 mM sodium chloride 25 mM Hepes buffer (pH 7.4). Fractions (0.5 ml) were collected and the most concentrated vesicle fractions combined.

### Liposomal encapsulation of mitoxantrone

Mitoxantrone (2.0 mg $ml^{-1}$) was titrated to pH 7.4 using 0.1 M sodium hydroxide and an appropriate volume of DSPC/Chol LUVs exhibiting a transmembrane pH gradient ($pH4.0_{in}/pH7.4_{out}$) was added to achieve a drug to phospholipid molar ratio of 0.416:1. This mixture was adjusted to the desired final mitoxantrone concentration by addition of 150 mM sodium chloride, 25 mM Hepes pH 7.4 and incubated at 55°C for 60 min. In preliminary experiments to determine the kinetics of mitoxantrone uptake, aliquots (100 μl) of this mixture were taken at various times and unencapsulated drug removed by passage of the LUVs through a 1-ml Sephadex G-50 (medium) minicolumn (Pick, 1981).

### Release kinetics of liposomal mitoxantrone in vitro

Large unilamellar vesicles composed of DSPC/Chol (55:45 molar ratio) or DSPC/Chol/$DPPE-PEG_{2000}$ (50:45:5 molar ratio), and containing [$^3$H]DPPC as a lipid marker, were prepared as described earlier. Drug loading was achieved by incubation of mitoxantrone with the LUVs (0.416:1 drug – phospholipid molar ratio) at 55°C for 60 mins. The loaded liposomes were then transferred to dialysis tubing (Spectra/Por 2, mol. wt cut-off 12–14 000) and dialysed against 250 volumes of 150 mM sodium chloride 25 mM Hepes pH 7.4 at 37°C with constant stirring. At 24 h, the transmembrane pH gradient was collapsed by the addition of nigericin (25 nM) to both the contents of the dialysis bag and the external solution. At various times up to 120 h, aliquots were removed from the dialysis bag and assayed for lipid (liquid scintillation counting) and mitoxantrone (see spectrophotometric assay below).

### Plasma clearance and biodistribution study

Large unilamellar vesicles composed of either DSPC/Chol (55:45) or DSPC/Chol/$DPPE-PEG_{2000}$ (50:45:5) were prepared as described earlier but containing the non-exchangeable, non-metabolizable lipid marker [$^3$H]cholesterol hexadecyl ether (Huang, 1983). Mitoxantrone was loaded as described earlier at a drug – phospholipid molar ratio of 0.416:1, employing [$^{14}$C]mitoxantrone as a radiolabelled marker.

The plasma clearance and tissue distribution of free mitoxantrone, mitoxantrone-loaded DSPC/Chol liposomes, control 'empty' DSPC/Chol liposomes, mitoxantrone-loaded DSPC/Chol/DPPE-$PEG_{2000}$ liposomes and control 'empty' DSPC/Chol/$DPPE-PEG_{2000}$ liposomes, were determined in female BDF1 mice (mean body weight, 20 g). For the free drug and both liposomal mitoxantrone formulations, single intravenous doses were administered via a lateral tail vein at 10 mg $kg^{-1}$ mitoxantrone in an injection volume of 200 μl. Control liposomes (no mitoxantrone) were administered at the same phospholipid dose as that of the drug-loaded vesicles. At 1, 4, 24 and 72 h, three animals from each experimental group were anaesthetized (160 mg $kg^{-1}$ Ketamine, 20 mg $kg^{-1}$ xylazine) and blood samples taken by heart puncture using a 25-gauge needle. Blood (approximately 200 μl) was collected into 'Microtainer' tubes containing EDTA and placed on ice. Animals were then sacrificed by cervical dislocation and dissected to remove the lungs, liver, spleen, kidney and muscle.

Liposomal lipid and mitoxantrone concentrations in plasma were determined by dual-label liquid scintillation counting (Beckman LS3801 instrument) with a detection limit set of 50 d.p.m. over background. Plasma samples containing high concentrations of mitoxantrone were first decolorized with 30% hydrogen peroxide. Plasma and tissue levels of mitoxantrone given under Results will include any radiolabelled metabolites. In the case of liposomal mitoxantrone, plasma drug levels can be considered to represent encapsulated mitoxantrone in view of the very rapid clearance of free drug from this compartment (see Results).

Organs and tissues were weighed and a 10% homogenate prepared in distilled water using a Brinkmann Polytron homogenizer.

© Cancer Research Campaign 1997

An aliquot (200 µl) of the homogenized sample was then digested with 500 µl of Solvable at 50°C for 3 h. After cooling, EDTA (50 µl of a 200 mM stock solution) was added followed by 30% hydrogen peroxide (200 µl) and finally 10 M hydrogen chloride (25 µl). After incubation at room temperature for 1 h, scintillation cocktail was added and the samples then kept in the dark overnight before determining liposomal lipid and mitoxantrone levels using dual-label liquid scintillation counting. A plasma correction factor was used in the calculation of tissue liposome and drug levels.

Trapezoidal area under the curve (AUC) calculations were based on average plasma and tissue levels of mitoxantrone and liposomal lipid at the indicated time points.

### Anti-tumour efficacy study

Female BDF1 mice were inoculated intravenously with $10^4$ L1210 murine tumour cells, derived from the ascites fluid of a previously infected BDF1 mouse. Free mitoxantrone or mitoxantrone encapsulated in either DSPC/Chol or DSPC/Chol/DPPE-$PEG_{2000}$ liposomes was administered via a lateral tail vein, 24 h after tumour cell inoculation. Animal weights were monitored daily for 14 days and mortality determined up to 60 days. Mean and median survival times and percentage increase in life span (%ILS) of treated animals compared with untreated controls, were calculated. In addition, liposomal – free (L/F) survival time ratio values, which reflect the anti-cancer potency of liposomal mitoxantrone compared with free mitoxantrone, at the same dose, were determined.

### Analytical procedures

In some experiments, mitoxantrone was quantified using a spectrophotometric assay. An aliquot of the liposomal drug was diluted with distilled water to a volume of 300 µl and 1200 µl of 5% Triton X-100 (w/v) then added. The mixture was heated at 60°C for 5 min to release all the encapsulated mitoxantrone and absorbance then read at 666.5 nm. Drug concentration in the sample was then determined by comparison to a standard curve (0–60 nmol mitoxantrone). This spectroscopic assay was compared with the use of [$^{14}$C]mitoxantrone to determine mitoxantrone uptake and release in vitro and for determination of drug levels in plasma. Both assays gave similar results.

Vesicle size distributions were determined by quasielastic light scattering using a Nicomp 270 submicron particle sizer as described previously (Madden et al, 1988). Phospholipid concentrations were determined by phosphate assay after perchloric acid digestion (Fiske and Subbarow, 1925).

### RESULTS

Academic and clinical studies of liposomal anti-cancer drugs have been greatly facilitated by the development of a remote-loading procedure that allows efficient drug encapsulation into preformed liposomes. This technique takes advantage of drug redistribution in response to a transmembrane pH gradient (Mayer et al, 1986*b*, Cullis et al, 1989; Madden et al, 1990). As shown in Figure 1, when mitoxantrone is incubated at 55°C with DSPC/Chol liposomes

**Figure 1** Uptake of mitoxantrone by DSPC/Chol (55:45 mol/mol) liposomes. Mitoxantrone was loaded at a drug to phospholipid molar ratio of 0.416:1 into vesicles exhibiting a transmembrane pH gradient ($pH4.0_{in}/pH7.4_{out}$) at 23°C (○) and (●) at 55°C Mitoxantrone was assayed as indicated under Materials and methods using the spectrophotometric procedure whereas DSPC was quantitated by phosphate assay

**Figure 2** Release of mitoxantrone from DSPC/Chol (55:45 mol/mol) liposomes (○) and DSPC/Chol/DPPE-$PEG_{2000}$ (50:45:5) liposomes (●) under dialysis conditions at 37°C. Mitoxantrone was loaded at a drug to phospholipid molar ratio of 0.416:1 and at 1.0 mg ml$^{-1}$. Nigericin (25 nM) was added at 24 hs. Mitoxantrone ([$^{14}$C]mitoxantrone) and phospholipid ([$^{3}$H] DPPC) were determined by liquid scintillation counting as described under Materials and methods

**Table 1** Mean area under the curve (AUC) for mitoxantrone and liposomal lipid in plasma and selected tissues: comparison of conventional and sterically stabilized liposomes

| | Plasma (μmol h ml$^{-1}$) | | Liver (μmol h g$^{-1}$) | | Spleen (μmol h g$^{-1}$) | | Lung (μmol h g$^{-1}$) | | Kidney (μmol h g$^{-1}$) | |
|---|---|---|---|---|---|---|---|---|---|---|
| | $AUC_{PL}$ | $AUC_{MITO}$ | $AUC_{PL}$ | $AUC_{MITO}$ | $AUC_{PL}$ | $AUC_{MITO}$ | $AUC_{PL}$ | $AUC_{MITO}$ | $AUC_{PL}$ | $AUC_{MITO}$ |
| DSPC/Chol | 11.51 | 4.44 | 28.88 | 10.88 | 79.83 | 28.44 | 1.08 | 0.40 | 3.96 | 1.69 |
| DSPC/Chol/PEG-PE | 28.27 | 10.95 | 27.20 | 9.69 | 45.71 | 16.31 | 2.02 | 0.49 | 4.11 | 1.57 |

exhibiting a pH gradient, rapid drug encapsulation is observed. Essentially, complete uptake occurs within 60 min and this uptake is stable with no loss of accumulated drug seen up to 3 h at this temperature. In addition to being dependent on the imposed pH gradient, this uptake is highly temperature sensitive. As shown in Figure 1, very little accumulation is seen for vesicles incubated with drug at room temperature. This probably reflects the temperature dependence of the permeability coefficient for DSPC/Chol bilayers and is consistent with previous reports on liposomal uptake of other anti-cancer agents (Mayer et al, 1986*b*). Liposomes composed of DSPC/Chol/DPPE-$PEG_{2000}$ exhibiting a pH gradient ($pH4.0_{in}pH7.4_{out}$) also showed essentially complete uptake of mitoxantrone at 55°C.

Before comparing the pharmacokinetic and therapeutic properties of mitoxantrone in conventional or sterically stabilized liposomes, it was important to determine whether the incorporation of DPPE-$PEG_{2000}$ influenced the physical properties of the liposomal carrier. In particular we examined whether this PEG – lipid conjugate increased bilayer permeability and hence the rate of release of encapsulated drug. Mitoxantrone, therefore, was loaded into DSPC/Chol and DSPC/Chol/DPPE-$PEG_{2000}$ liposomes and these vesicles dialysed against Hepes-buffered saline at 37°C. Further, after 24 h the residual transmembrane pH gradient was dissipated by addition of the ionophore nigericin, and dialysis continued for an additional 96 h. The stability of mitoxantrone-loaded liposomes is well illustrated by the data presented in Figure 2. For liposomal systems with or without DPPE-$PEG_{2000}$, no measurable drug loss was seen over 24 h. Even after dissipation of the residual pH gradient, only very slow leakage is observed with approximately 85% of the initially encapsulated mitoxantrone retained at 120 h for both DSPC/Chol and DSPC/Chol/DPPE-$PEG_{2000}$ liposomes. This slow drug release is surprising, particularly given that dissipation of the pH gradient will not only remove the driving force behind drug redistribution, but will also raise the intravesicular pH, thereby increasing the proportion of mitoxantrone present as the neutral, membrane permeant species. Clearly, even at 37°C, the permeability coefficient of DSPC/Chol bilayers to mitoxantrone is relatively low. This point will also be addressed in the Discussion.

The behaviour of drug-loaded liposomes in vivo was then examined using a murine model. Plasma clearance rates of mitoxantrone and liposomal lipid following intravenous administration of free drug or mitoxantrone encapsulated in either DSPC/Chol or DSPC/Chol/DPPE-$PEG_{2000}$ liposomes, are shown in Figure 3. In contrast to the rapid clearance seen for free drug, over 90% of the administered mitoxantrone remained in the circulation at 1 h when given in a liposomal carrier (Figure 3A). Comparison of the two liposome formulations indicates, as expected, that the conventional systems are cleared from the circulation more rapidly than those containing DPPE-$PEG_{2000}$. For both formulations very little drug or lipid remained in the blood at 72 h (Figure 3A and B). An indication of mitoxantrone leakage from the carrier can be obtained from the calculation of drug-to-lipid ratio for those liposomes remaining in the circulation at various time points. As shown in Figure 3C, up to 24 h, this ratio remains similar to the initial value, particularly for the sterically stabilized liposomes, indicating little drug

**Figure 3** Plasma concentrations of mitoxantrone (**A**) and liposomal phospholipid (**B**) as well as circulating mitoxantrone to phospholipid molar ratio × 10 (**C**) in mice injected i.v. with free mitoxantrone (◆), mitoxantrone entrapped in DSPC/Chol (55:45 mol/mol) (●), and DSPC/Chol/DPPE-$PEG_{2000}$ (50:45:5) (○) liposomes. The mitoxantrone dose was 10 mg kg$^{-1}$; the phospholipid dose was 54 μmol kg$^{-1}$; initial mitoxantrone to phospholipid molar ratio was 0.416. Mitoxantrone ([$^{14}$C]mitoxantrone) and phospholipid ([$^{3}$H]CHE) were assayed as described under Materials and methods. Mean ± s.e.m., *n*=3

© Cancer Research Campaign 1997

**Figure 4** Distribution of mitoxantrone and liposomal lipid in mice injected i.v. with DSPC/Chol (55:45 mol mol) liposomal mitoxantrone (●) and DSPC/Chol/DPPE-PEG$_{2000}$ (50:45:5) liposomal mitoxantrone (○) at various times post injection. Plasma (**A**), liver (**B**) and spleen (**C**) drug and lipid levels are shown. Mice received 10 mg kg$^{-1}$ mitoxantrone and 54 μmol kg$^{-1}$ phospholipid. Tissues were prepared and mitoxantrone ([$^{14}$C] mitoxantrone) and phospholipid ([$^{3}$H]-CHE) were assayed as described in Materials and methods. Mean ± s.e.m. $n = 3$. Data shown have been corrected for drug or lipid in the blood compartment of each tissue

© Cancer Research Campaign 1997

**Figure 5** Tissue distribution in lung (**A**) and kidney (**B**) of mitoxantrone and liposomal lipid in mice injected i.v. with DSPC/Chol (55:45 mol/mol) liposomal mitoxantrone (●) and DSPC/Chol/DPPE-PEG$_{2000}$ (50:45:5) liposomal mitoxantrone (○) at various times post injection. Mice received 10 mg kg$^{-1}$ mitoxantrone and 54 µmol kg$^{-1}$ phospholipid. Tissues were prepared and mitoxantrone [$^{14}$C]mitoxantrone) and phospholipid ([$^{3}$H]CHE) were assayed as described in Materials and methods. Mean ± s.e.m. $n$ = 3. Data shown have been corrected for drug or lipid in the blood compartment of each tissue leakage. Between 24 and 72 h, however, vesicles remaining in the circulation appear to lose most of their drug load.

We also examined the tissue distribution of mitoxantrone and liposomal lipid at the same time points used in the plasma study. Liver accumulation of the drug and carrier following administration of DSPC/Chol systems was significantly different from that of DSPC/Chol/DPPE-PEG$_{2000}$ liposomes only at the 1- and 4-h time points. Despite the fact that plasma levels at 24 h were fivefold greater for the DSPC/Chol/DPPE-PEG$_{2000}$ vesicles compared with the conventional carriers, no significant differences were seen in liver levels at either 24 or 48 h. In contrast, spleen accumulation of lipid and drug was reduced at all time points evaluated for the sterically stabilized carriers. These clearance data (Figure 4) are reflected in the mean area under the curve (AUC) analysis shown in Table 1. Mitoxantrone and liposomal lipid exposure in the liver was comparable for the two formulations studied, whereas spleen exposure was reduced when using the DSPC/Chol/DPPE-PEG$_{2000}$ formulation. This result suggests that incorporation of PEG-conjugated lipids into the liposomal carriers studied here inhibits recognition and clearance within the spleen more effectively than within the liver. As would be expected, mitoxantrone and liposomal lipid AUC values in plasma are greater for the sterically stabilized vesicles than conventional liposomes.

Mitoxantrone and liposomal lipid accumulations within the kidney, lung and muscle (rectus femoris) were also determined for both liposomal formulations. As shown in Figure 4, drug and lipid levels in lung and kidney were considerably lower than for liver and spleen and in the case of muscle were essentially below detectable limits (<2 p mol g$^{-1}$).

It is clear from the data presented in Figures 4 and 5 that a close correlation exists between tissue levels of mitoxantrone and liposomal lipid. This observation can be most readily accounted for if

© Cancer Research Campaign 1997

**Table 2** Anti-tumour efficacy of mitoxantrone in conventional and sterically stabilized liposomes against a murine leukemia cell line (L1210) in BDF1 mice. Mice were inoculated with 10 000 L1210 cells i.v. on day zero and treatment initiated on day 1

| Treatment group | Dose (mg kg$^{-1}$) | No. of animals | Average weight change (% day 10) | No. of survivors (day 40) | Mean survival[a] Time | Mean survival[a] Time | %ILS[2] | L/F[b] | Mean survival (post-reinoculation)[c] |
|---|---|---|---|---|---|---|---|---|---|
| Control | 0 | 10 | NA | 0/10 | 7.7 | NA | | | |
| Free | 5 | 5 | 0.5 | 0/5 | 12.6 | 13 | 63 | | |
| Free | 10 | 5 | –12.2 | 0/5 | 17.6 | 15 | 88 | | |
| DSPC | 5 | 5 | 3.9 | 0/5 | 11.0 | 11 | 38 | 0.85 | |
| DSPC | 10 | 10 | –1.1 | 0/10 | 14.2 | 14.5 | 81 | 0.97 | |
| DSPC | 20 | 10 | –12.3 | 4/10 | 30.7 | 23.5 | 194 | | 8 |
| DSPC | 30 | 10 | –21.4 | 1/10 | 19.8 | 15 | 88 | | 8 |
| DSPC-PEG | 10 | 5 | –0.9 | 0/5 | 12.8 | 13 | 63 | 0.87 | |
| DSPC-PEG | 20 | 5 | –7.0 | 1/5 | 25.4 | 23 | 188 | | |

[a]To calculate mean and medial survival time, survivors after 40 days were assigned survival times of 40 days. [b]Values for percentage increase in life span (%ILS) and liposomal/free (L/F) were calculated using median survival data. [c]Reinoculation of survivors was on day 63 with 10 000 L1210 cells i.v. per survivor.

tissue mitoxantrone content reflects deposition of drug-loaded liposomes. Further, this observation would imply that, once accumulated within a tissue, only slow drug release occurs from the liposomal carrier.

The anti-cancer efficacies of free mitoxantrone and mitoxantrone encapsulated in DSPC/Chol or DSPC/Chol/DPPE-PEG$_{2000}$ liposomes were then compared in a murine tumour model. As shown in Table 2, administration of free drug at 5 or 10 mg kg$^{-1}$ produces a dose-dependent increase in survival time relative to the untreated control group. Higher doses of free drug (15 or 20 mg kg$^{-1}$) were found to be toxic, resulting in mortality at earlier time points than seen for control animals. When mitoxantrone is administered within liposomes, however, its toxicity is significantly ameliorated (Table 2). Whereas free drug at 10 mg kg$^{-1}$ produces a weight loss of about 12% at the nadir (day 10), an equivalent dose of mitoxantrone in DSPC/Chol or DSPC/Chol/DPPE-PEG$_{2000}$ vesicles results in no significant weight change. Even at 20 mg kg$^{-1}$ the liposomal formulations produce a mean percentage weight loss of less than 5%. Further escalation in liposomal drug dose to 30 mg kg$^{-1}$, however, does elicit drug-related deaths.

A comparison of the percentage increase in lifespan (%ILS) produced by 5 or 10 mg kg$^{-1}$ free or liposomal mitoxantrone indicates that at equivalent doses the liposomal drug exhibits similar or slightly lower efficacy. This is illustrated by the liposome – free ratio of 0.85 (5 mg kg$^{-1}$) and 1.00 (10 mg kg$^{-1}$). The lower toxicities of the liposomal formulations, however, allow treatment at higher doses and at 20 mg kg$^{-1}$ mitoxantrone, in either DSPC/Chol or DSPC/Chol/DPPE-PEG$_{2000}$ vesicles, %ILS values are significantly greater than seen for free drug at the MTD. Interestingly, there does not appear to be an improvement in efficacy for the sterically stabilized vesicles compared with conventional liposomes. At 30 mg kg$^{-1}$ mitoxantrone in DSPC/Chol liposomes the %ILS is lower than for the same formulation administered at 20 mg kg$^{-1}$. This reflects drug-induced toxicity as indicated by animal weight loss data.

In addition to the improvement in %ILS relative to free drug at the MTD, survivors were obtained in the groups treated with liposomal mitoxantrone at 20 mg kg$^{-1}$ and 30 mg kg$^{-1}$. To confirm that these survivors were not the result of an immunological response to the tumour cell line, animals were reinoculated with the same L1210 tumour line and not subsequently treated. As shown in Table 2, these survivors succumbed over the same time frame as control animals.

We strongly believe that this anti-tumour activity results from the antiproliferative activity of mitoxantrone rather than inhibition of tumour cell seeding. This interpretation is based on the following considerations. The L1210 cell line exhibits a doubling time of approximately 12 h in vitro and it is likely therefore that the cells are well established in the tissue 24 h after intravenous injection, at which time therapy is initiated. Histological studies of livers obtained from animals 3 days after L1210 inoculation confirm that there is diffuse infiltration of tumour cells throughout the tissue. Further, when animals were administered DSPC/Chol liposomes (no mitoxantrone) at a dose of 100 mg ml$^{-1}$ 1 day after i.v. administration of L1210 cells, no difference in survival was seen compared with untreated controls.

### DISCUSSION

Earlier studies demonstrating that liposomes can selectively accumulate at sites of infection, inflammation and neoplastic disease provided both a rationale for their use as drug carriers and an explanation for any observed changes in drug toxicity or efficacy (Morgan, et al, 1985; Ogihara et al, 1986). Particularly in the case of highly cytotoxic antineoplastic agents, the ability to enhance tumour drug levels while reducing exposure to healthy tissues and organs would be expected to significantly improve the agent's therapeutic index. This expectation has been confirmed by animal studies showing that the toxicities of doxorubicin, vincristine and other anti-cancer drugs, including mitoxantrone, can be significantly reduced by administration within a liposomal carrier (Gabizon et al, 1982; Olson et al, 1982; Mayer et al, 1989; Mayer et al, 1990*a*; Schwendener et al, 1991). Similar benefits have been reported in human clinical trials, most notably a marked reduction in cardiotoxicity for liposomal doxorubicin (Batist et al, 1992; Northfield et al, 1993). The present results are consistent with this earlier research and confirm that liposomal incorporation of mitoxantrone results in a marked reduction in drug toxicity. In turn, this allows administration of larger and more efficacious drug doses.

In addition to the benefits resulting from reduced toxicity, liposomal encapsulation has been shown to enhance drug delivery to solid or ascitic tumours and this can result in improved efficacy

© Cancer Research Campaign 1997 

(Gabizon and Papahadjopoulos, 1988; Mayer et al, 1990*b*; Gabizon, 1992; Huang et al, 1992; Bally et al, 1994). This selective accumulation is believed to reflect extravasation of the liposomal carrier via leaky, immature capillary endothelium formed as the result of rapid angiogenesis within the growing tumour (Bally et al, 1994). A number of factors would be expected to influence the extent of this tumour localization, including liposome size, composition and circulation half-life. Given that conventional liposomes are cleared from the blood fairly rapidly by phagocytic cells of the reticuloendothelial system (RES), considerable research has been directed towards the development of systems that avoid recognition and uptake by the RES. This has been achieved by incorporating into the liposomal membrane lipids possessing relatively bulky polar headgroups, such as $G_{M1}$ or phosphatidylinositol (Gabizon and Papahadjopoulos, 1988; Gabizon et al, 1989), or phospholipids conjugated to a hydrophilic polymer such as poly(ethylene glycol) (Klibanov et al, 1990; Allen et al, 1991). Such sterically stabilized liposomes exhibit longer circulation half-lives than equivalent conventional systems and this has been shown to result in greater accumulation at tumour sites (Papahadjopoulos et al, 1991; Huang et al, 1992; Vaage et al, 1992; Maruyama et al, 1994).

Previous studies on liposomal mitoxantrone have employed systems in which the cationic drug is associated with liposomes containing an acidic phospholipid, phosphatidic acid, via predominantly electrostatic interactions (Schwendener et al, 1991). Following intravenous administration of these systems, mitoxantrone is rapidly cleared from the blood with an initial elimination half-life of less than 30 min. This may reflect either rapid drug loss from the carrier or recognition and clearance of the negatively charged liposomes by the RES. More recently this same group has reported the pharmacokinetics of mitoxantrone encapsulated in sterically stabilized liposomes composed of soy phosphatidylcholine (SPC) and cholesterol (Schwendener et al, 1994). Surprisingly, despite the use of a pH gradient for drug loading, fairly rapid loss of mitoxantrone from the carrier was observed with approximately 95% of the injected dose cleared from the circulation within 2 h. In the present research, therefore, we have encapsulated mitoxantrone within neutral liposomes composed of a saturated phosphatidylcholine (DSPC) and cholesterol. These systems show extended blood residency times. In the case of sterically stabilized liposomes containing a PEG – lipid conjugate, for example, more than 80% of the lipid and drug are present in the blood 4 h after injection and, even at 24 h, more than 30% remain in the circulation.

Whereas enhanced circulation lifetimes may result in greater tumour sequestration of liposomal drugs (Allen, 1994), this does not necessarily result in a corresponding increase in drug efficacy. Sterically stabilized liposomes containing mitoxantrone exhibit much longer circulation lifetimes than similar systems without a PEG – lipid conjugate but show no improvement in anti-cancer efficacy. As discussed recently (Mayer et al, 1994) this observation illustrates the fact that several factors influence the activity of liposomal drugs in vivo. Following localization at the tumour site, for example, it appears unlikely that the drug carrier is directly internalized by tumour cells. Instead it is believed that drug release occurs via passive diffusion across the liposomal bilayer or as the result of carrier processing by macrophages. Free drug would then be taken up into tumour cells via normal transport mechanisms (Allen, 1994). The mitoxantrone-loaded liposomes used in the present study, however, appear to be exceptionally stable. Following pH-driven encapsulation at 55°C, no drug release was observed over 24 h at 37°C and, even when the pH gradient was collapsed by the addition of nigericin, only low levels of mitoxantrone leakage occurred over a subsequent 96-h period. While these in vitro data cannot be directly related to the behaviour of these liposomal systems in vivo, taken together with the slow rate of drug release observed from liposomes in the blood and the strong correlation between mitoxantrone and liposomal lipid biodistribution, it suggests that, following accumulation within tumour sites, mitoxantrone release from the carrier may represent the limiting factor in determining drug efficacy.

In conclusion, we have shown that liposomal encapsulation significantly reduces mitoxantrone toxicity. This, in turn, allows administration of higher drug doses, resulting in improved efficacy. In addition, we have demonstrated that sterically stabilized liposomes containing mitoxantrone exhibit extended blood residency times compared with conventional systems but that this does not confer additional benefit with respect to drug efficacy. This latter observation may reflect a slow or incomplete release of entrapped mitoxantrone and we are, therefore, presently developing liposomal systems that will ensure full drug bioavailability following carrier deposition within the tumour.

### ACKNOWLEDGEMENTS

This research was supported by a grant from the National Cancer Institute of Canada with funds from the Canadian Cancer Society.

### REFERENCES

Allen TM (1994) Long circulating (sterically stabilized) liposomes for targeted drug delivery. *Trends Pharm Sci* **15:** 215–220

Allen TM, Hansen C, Martin F, Redemann C and Yan-young A (1991) Liposomes containing synthetic lipid derivatives of polyethylene glycol show prolonged circulation half-lives *in vivo*. *Biochim Biophys Acta* **1066:** 29–36

Bally MB, Masin D, Nayar R, Cullis PR and Mayer LD (1994) Transfer of liposomal drug carriers from the blood to the peritoneal cavity of normal and ascitic tumour-bearing mice. *Cancer Chemother Pharmacol* **34:** 137–146

Batist G, Panasci L, Gruner P, Leyland-Jones P, Pilkiewicz F and Haccoun L (1992) Phase II study of liposomal doxorubicin (TLC D-99) in metastatic breast cancer. *Proc Am Soc Clin Oncol* **11:** 82

Boman NL, Masin D, Mayer LD, Cullis PR and Bally MB (1994) Liposomal vincristine which exhibits increased drug retention and increased circulation longevity cures mice bearing P388 tumor. *Cancer Res* **54:** 2830–2833

Chonn A, Semple SC and Cullis PR (1992) Association of blood proteins with large unilamellar liposomes *in vivo*. Relation to circulation lifetimes. *J Biol Chem* **267:** 18759–18765

Cullis PR, Mayer LD, Bally MB, Madden TD and Hope MJ (1989) Generating and loading of liposomal systems for drug-delivery applications. *Adv Drug Del Rev* **3:** 267–282

Fiske CM and Subbarow Y. (1925) The colourimetric determination of phosphorus. *J Biol Chem* **66:** 375–400

Forseen EA (1988) Chemotherapy with anthracycline liposomes. In *Liposomes as Drug Carriers*, Gregoriadis G (ed.) pp. 355–364. John Wiley and Sons: Chichester.

Funalo K, Yoda R and Kiwada H (1992) Contribution of complement system to destabilization of liposomes composed of hydrogenated egg phosphatidylcholine in rat fresh plasma. *Biochim Biophys Acta* **1103:** 198–204

Gabizon AA (1992) Selective tumor localization and improved therapeutic index of anthracyclines encapsulated in long-circulating liposomes. *Cancer Res* **52:** 891–896

Gabizon A, Dagan A, Goren D, Barenholz Y and Fuks Z (1982) Liposomes as *in vivo* carriers of adriamycin: reduced cardiac uptake and preserved antitumor activity in mice. *Cancer Res* **42:** 4734–4739

Gabizon A and Papahadjopoulos D (1988) Liposome formulations with prolonged circulation time in blood and enhanced uptake by tumors. *Proc Natl Acad Sci USA* **85:** 6949–6953.

© Cancer Research Campaign 1997

Gabizon A, Shiota R and Papahadjopoulos D (1989) Pharmacokinetics and tissue distribution of doxorubcin encapsulated in stable liposomes with long circulation times. *J Natl Cancer Inst* **81:** 1484–1488

Hope MJ, Bally MB, Webb G and Cullis PR (1985) Production of large unilamellar vesicles by a rapid extrusion procedure. Characterization of size distribution, trapped volume and ability to maintain a membrane potential. *Biochim Biophys Acta* **812:** 210–216

Huang L (1983) Liposome-cell interactions in vitro. In *Liposomes from Biophysics to Therapeutics*, Ostro MJ (ed.). Marcel Dekker: New York

Huang SK, Mayhew E, Gilani S, Lasic DD, Martin FJ and Papahadjopoulos D (1992) Pharmacokinetics and therapeutics of sterically stabilized liposomes in mice bearing C-26 colon carcinoma. *Cancer Res* **52:** 6774–6781

Klibanov AL, Maruyama K, Torchilin VP and Huang L (1990) Amphipathic polyethylene glycols effectively prolong the circulation time of liposomes. *Febs Lett* **268:** 235–237

Madden TD, Tilcock CPS, Wong K and Cullis PR (1988) Spontaneous vesiculation of large multilamellar vesicles composed of saturated phosphatidylcholine and phosphatidylglycerol mixtures. *Biochemistry* **27:** 8724–8730

Madden TD, Harrigan PR, Tai LCL, Bally MB, Mayer LD, Redelmeier TE, Loughrey HC, Tilcock CPS, Reinish LW and Cullis PR (1990) The accumulation of drugs within large unilamellar vesicles exhibiting a proton gradient: a survey. *Chem Phys Lipids* **53:** 37–46

Maruyama K, Unezaki S, Yuda T, Ishida O, Takahashi N, Suyinaka A, Huang L and Iwatsuru M (1994) Enhanced delivery and antitumor effect of doxorubicin encapsulated in long-circulation liposomes. *J Liposome Res* **4:** 143–165

Mayer LD, Hope MJ, Cullis PR and Janoff AJ (1986*a*) Solute distributions and trapping efficiencies observed in freeze-thawed multilamellar vesicles. *Biochem Biophys Acta* **817:** 193–200

Mayer LD, Bally MB and Cullis PR (1986*b*) Uptake of adriamycin into large unilamellar vesicles in response to a pH gradient. *Biochim Biophys Acta* **857:** 123–126

Mayer LD, Tai LCL, KO DSC, Masin D, Ginsberg RS, Cullis PR and Bally MB (1989) Influence of vesicle size, lipid composition and drug-to-lipid ratio on the biological activity of liposomal doxorubicin in mice. *Cancer Res* **49:** 5922–5930

Mayer LD, Bally MB, Loughrey H, Masin D and Cullis PR (1990a) Liposomal vincristine preparations which exhibit decreased drug toxicity and increased activity against murine L1210 and P388 tumors. *Cancer Res* **50:** 575–579

Mayer LD, Bally MB, Cullis PR, Wilson SL and Emerman JT (1990b) Comparison of free and liposome encapsulated doxorubicin tumor drug uptake and antitumor efficacy in the SC115 murine mammary tumor. *Cancer Lett* **53:** 183–190

Mayer LD, Cullis PR and Bally MB (1994) The use of transmembrane pH gradient-driven drug encapsulation in the pharmacodynamic evaluation of liposomal doxorubicin. *J Liposome Res* **4:** 529–553

Morgan JR, Williams LA and Howard CB (1985) Technetium-labelled liposome imaging for deep-seated infection. *Br J Radiol* **58:** 35–39

Northfield DW, Russel J, Anderson M, Lang J and Volberding PA (1993) Pharmacokinetics, tumor localization and safety of Doxil (liposomal doxorubicin) in AIDS patients with Karposi's sarcoma (AIDS-KS). *Proc Am Soc Clin Oncol* **12:** 51

Ogihara I, Kojimi S and Jay M (1986) Differential uptake of gallium-67-labelled liposomes between tumors and inflammatory lesions in rats. *J Nucl Med* **27:** 1300–1307

Olson F, Mayhew E, Maslow D, Rustum Y and Szoka F (1982) Characterization, toxicity and therapeutic efficacy of adriamycin encapsulated in liposomes. *Eur J Cancer Clin Oncol* **18:** 167–176

Papahadjopoulos D, Allen TM, Gabizon A, Mayhew E, Matthay K Huang SK, Lee KD, Woodle MC, Lasic DD, Redemann C and Martin FJ (1991) Sterically stabilized liposomes: improvements in pharmacokinetics and anti-tumor therapeutic efficacy. *Proc Natl Acad Sci USA* **88:** 11460–11464

Pestalozzi B, Schwendener R and Sauter C (1992) Phase I/II study of liposome-complexed mitoxantrone in patients with advanced breast cancer. *Ann Oncol* **3:** 445–449

Pick U (1981) Liposomes with a large trapping capacity prepared by freezing and thawing sonicated phospholipid mixtures. *Arch Biochim Biophys* **212:** 186–194

Presant CA, Proffitt RT, Smith JD and Mckenna RJ (1986) Evidence for solid tumor accumulation of intravenously injected lipid vesicles (LV) in patients. *Proc Am Acad Cancer Res* **27:** 158

Rahman A, Kessler A, More N, Sikic B, Rowden G, Woolley P and Schein PS (1980) Liposomal protection of adriamycin-induced cardiotoxicity in mice. *Cancer Res* **40:** 1532–1537

Schwendener RA, Fiebig HH, Berger MR and Berger DP (1991) Evaluation of incorporation characteristics of mitoxantrone into unilamellar liposomes and analysis of their pharmacokinetic properties, acute toxicity, and antitumor efficacy. *Cancer Chemother Pharmacol* **27:** 429–439

Schwendener RA, Horber DH, Rentsch K, Hanseler E, Pestalozzi B and Sauter C (1994) Preclinical and clinical experience with liposome-encapsulated mitoxantrone. *J Liposome Res* **4:** 605–639

Shenkenberg TD and Von Hoff DD (1986) Mitoxantrone: a new anticancer drug with significant clinical activity. *Ann Intern Med* **105:** 67–81

Smith IE (1983) Mitoxantrone (Novantrone): a review of experimental and early clinical studies. *Cancer Treat Rev* **10:** 103–115

Vaage J, Mayhew E, Lasic DD and Martin F (1992) Therapy of primary and metastatic mouse mammary carcinoma with doxorubicin encapsulated in long circulating liposomes. *Int J Cancer* **51:** 942–948

Williams BD, O'Sullivan MM, Saggu GS, Williams KE, Williams LA and Morgan JR (1986) Imaging in rheumatoid arthritis using liposomes labelled with technetium. *Br Med J* **293:** 1143–1144

© Cancer Research Campaign 1997


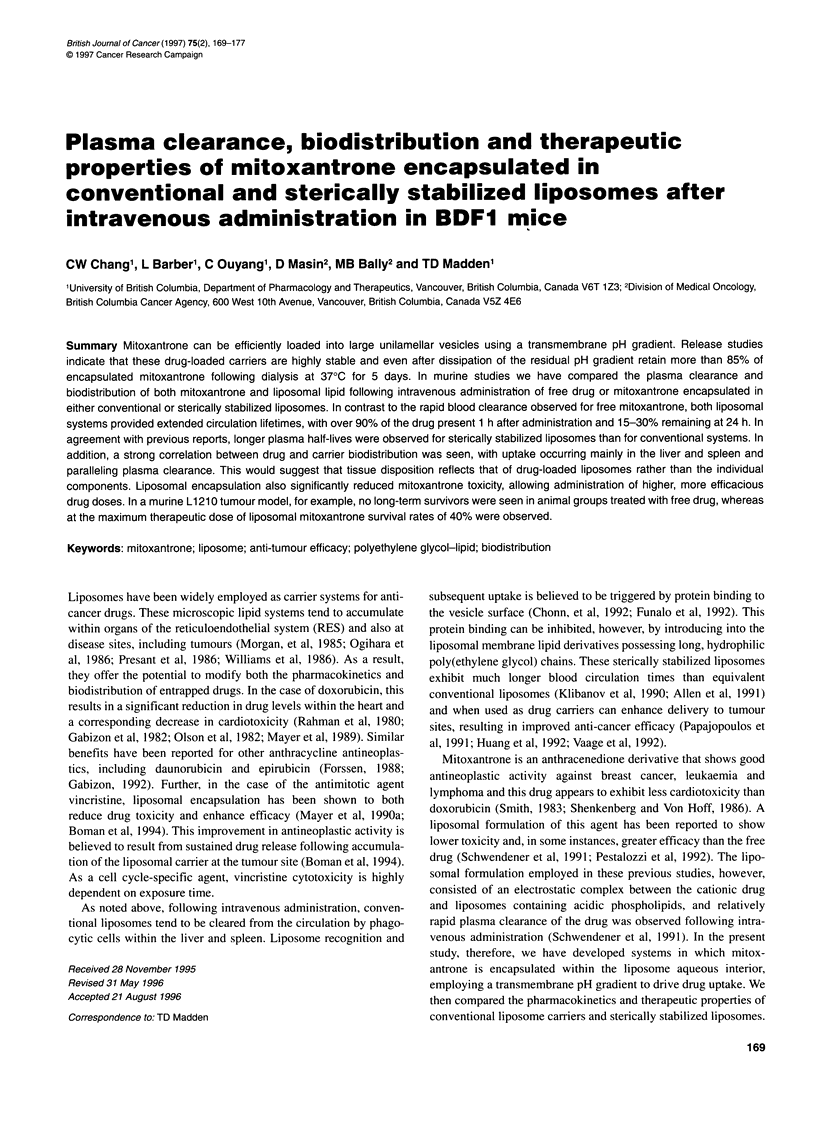

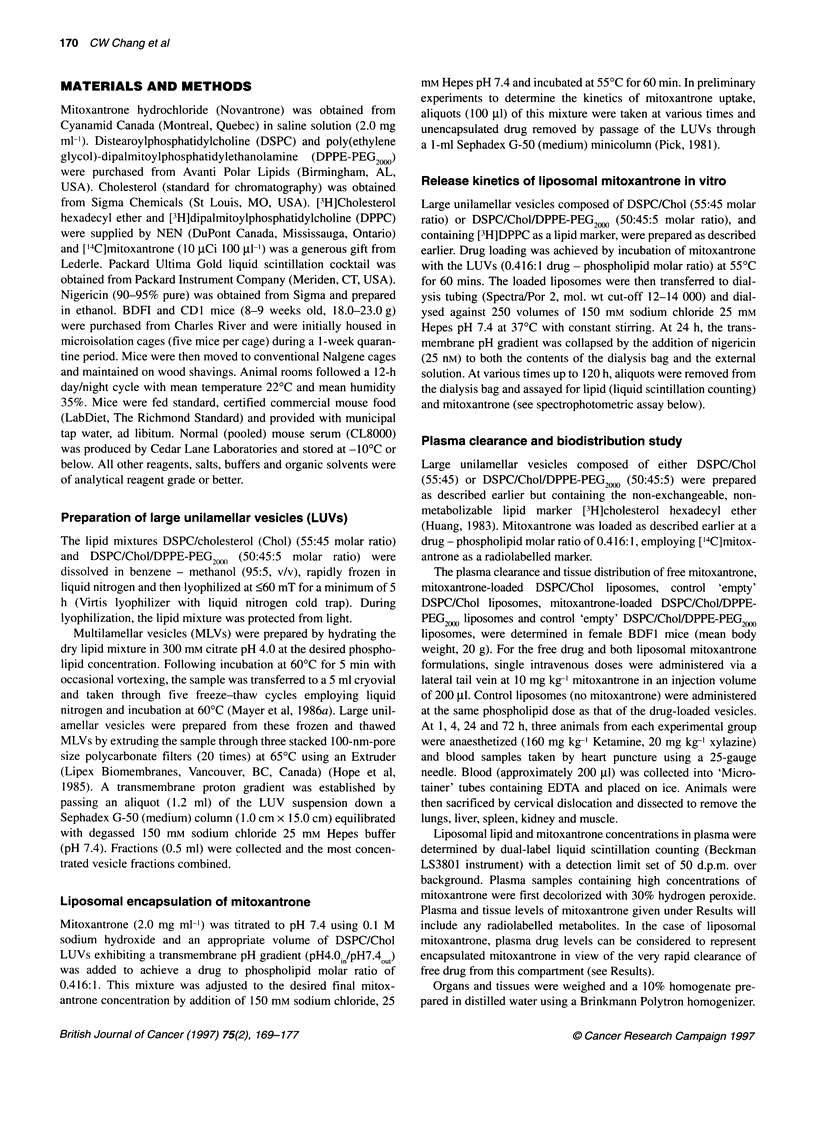

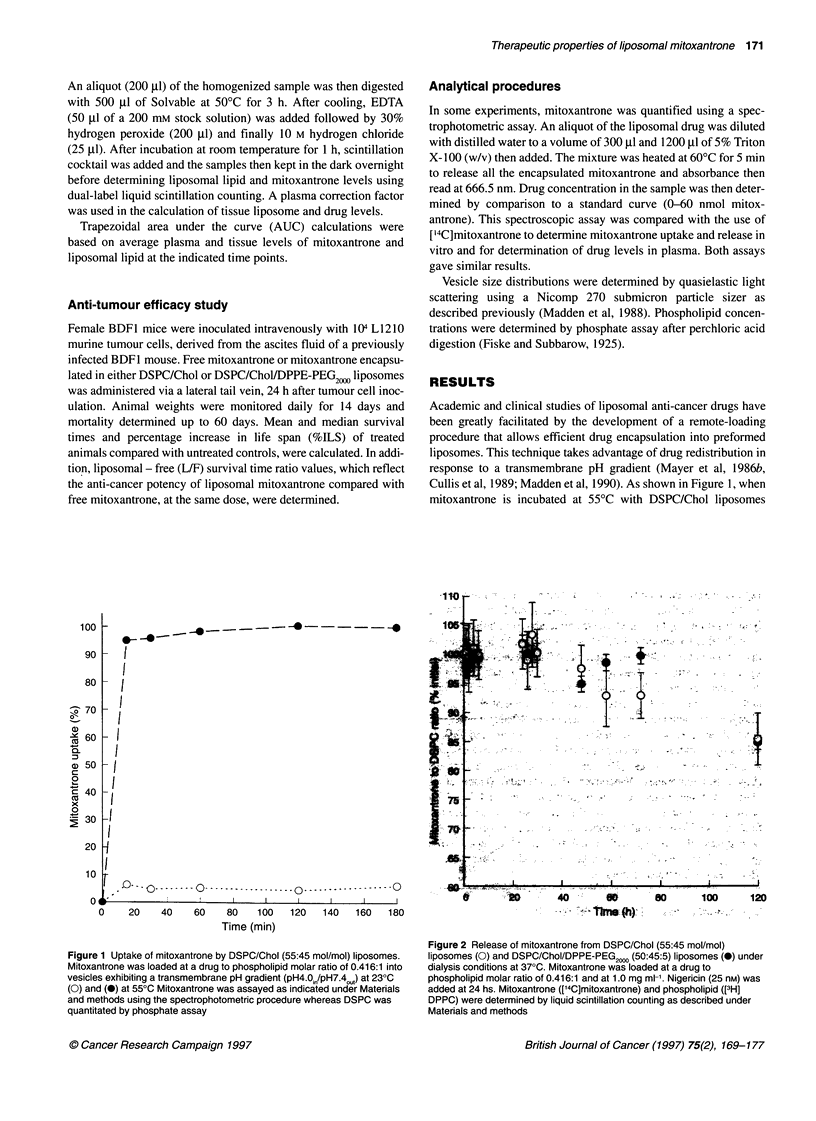

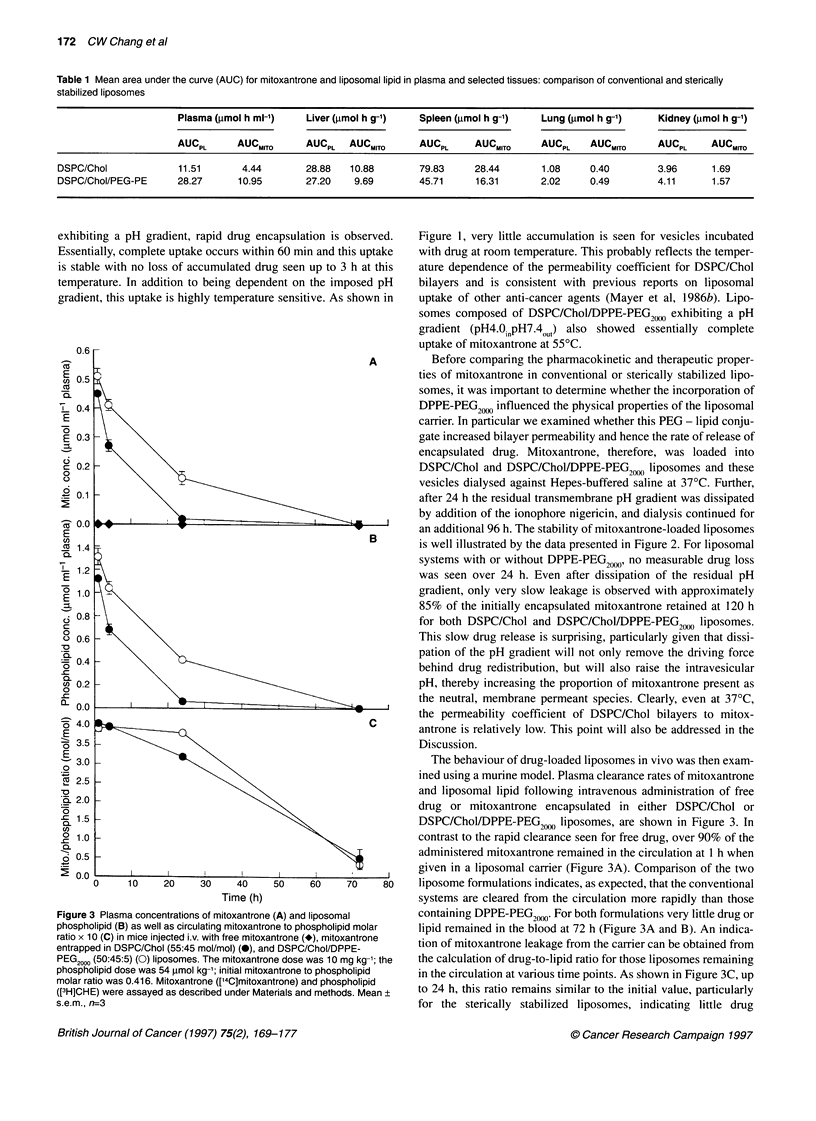

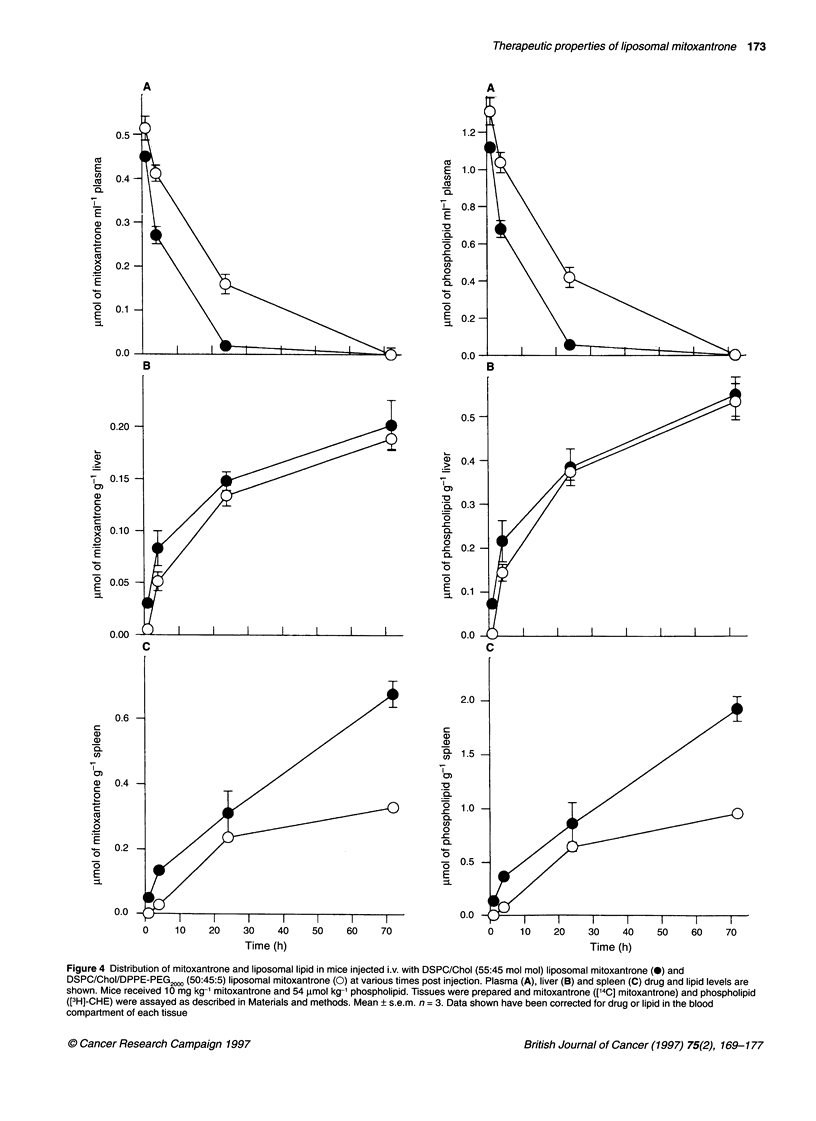

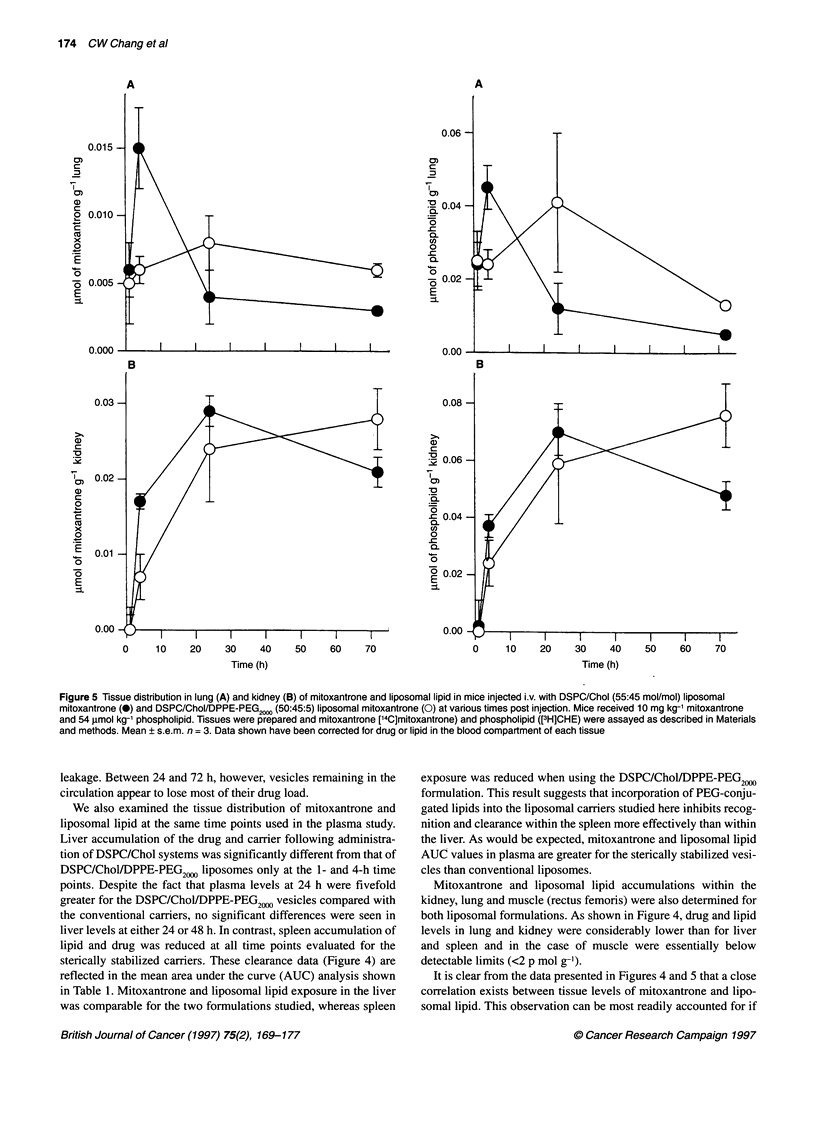

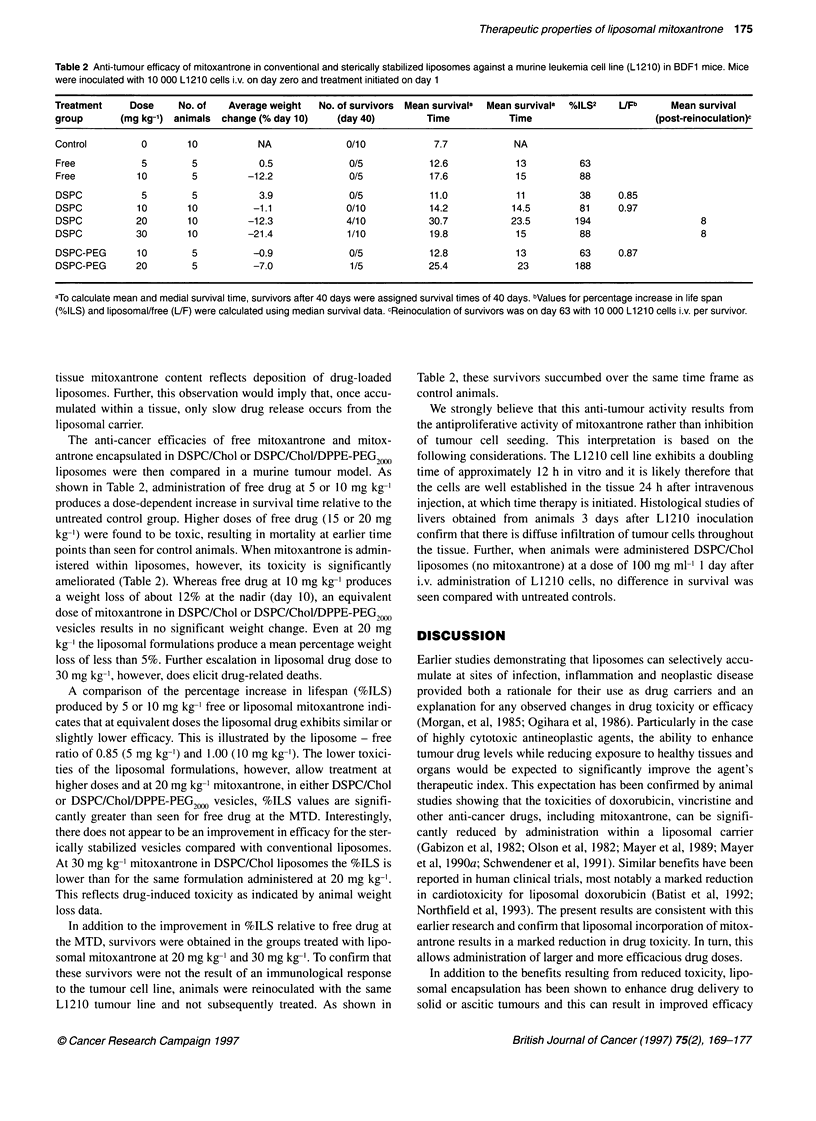

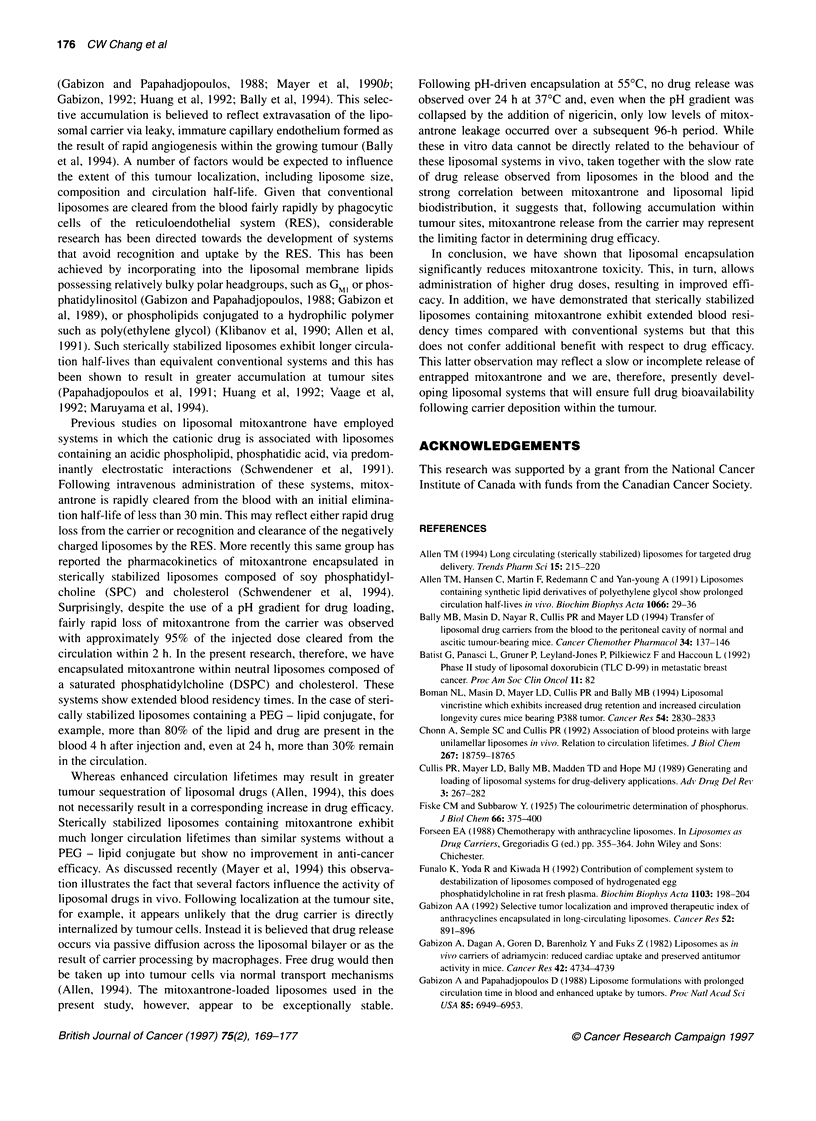

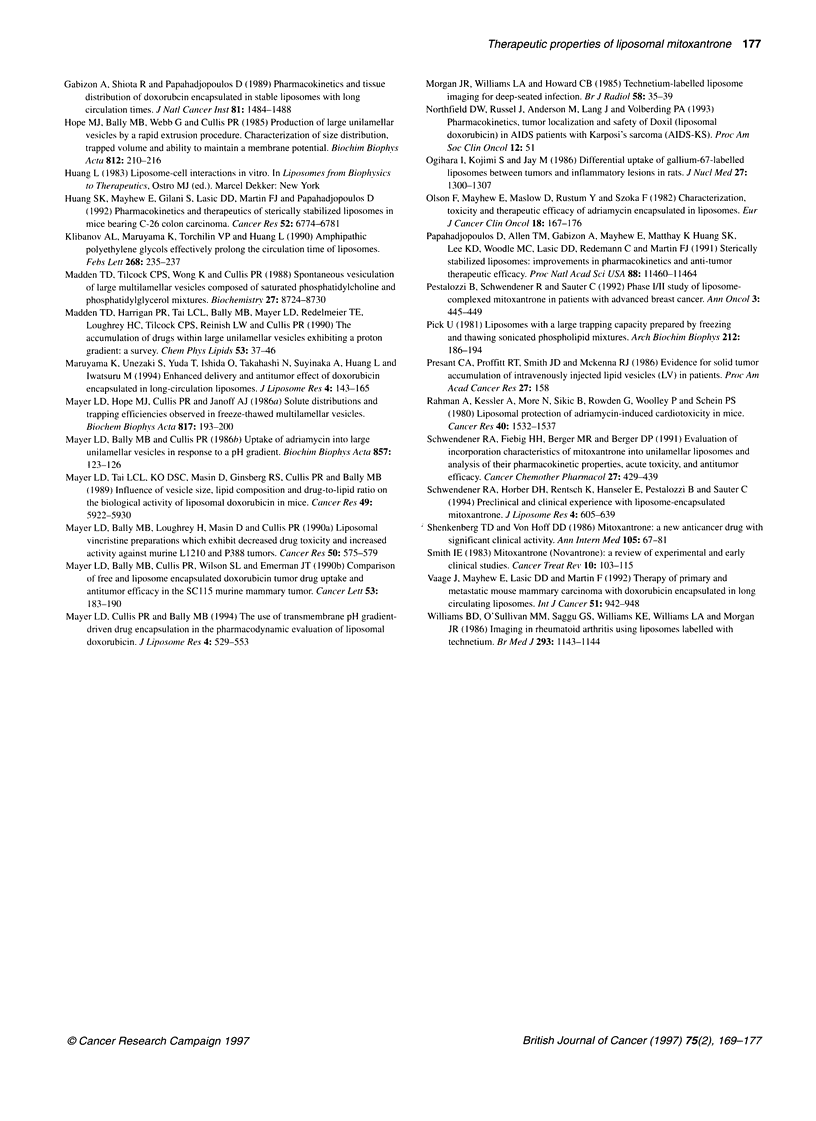


## References

[OCR_00996] Allen T. M., Hansen C., Martin F., Redemann C., Yau-Young A. (1991). Liposomes containing synthetic lipid derivatives of poly(ethylene glycol) show prolonged circulation half-lives in vivo.. Biochim Biophys Acta.

[OCR_00992] Allen T. M. (1994). Long-circulating (sterically stabilized) liposomes for targeted drug delivery.. Trends Pharmacol Sci.

[OCR_01001] Bally M. B., Masin D., Nayar R., Cullis P. R., Mayer L. D. (1994). Transfer of liposomal drug carriers from the blood to the peritoneal cavity of normal and ascitic tumor-bearing mice.. Cancer Chemother Pharmacol.

[OCR_01011] Boman N. L., Masin D., Mayer L. D., Cullis P. R., Bally M. B. (1994). Liposomal vincristine which exhibits increased drug retention and increased circulation longevity cures mice bearing P388 tumors.. Cancer Res.

[OCR_01016] Chonn A., Semple S. C., Cullis P. R. (1992). Association of blood proteins with large unilamellar liposomes in vivo. Relation to circulation lifetimes.. J Biol Chem.

[OCR_01035] Funato K., Yoda R., Kiwada H. (1992). Contribution of complement system on destabilization of liposomes composed of hydrogenated egg phosphatidylcholine in rat fresh plasma.. Biochim Biophys Acta.

[OCR_01040] Gabizon A. A. (1992). Selective tumor localization and improved therapeutic index of anthracyclines encapsulated in long-circulating liposomes.. Cancer Res.

[OCR_01045] Gabizon A., Dagan A., Goren D., Barenholz Y., Fuks Z. (1982). Liposomes as in vivo carriers of adriamycin: reduced cardiac uptake and preserved antitumor activity in mice.. Cancer Res.

[OCR_01050] Gabizon A., Papahadjopoulos D. (1988). Liposome formulations with prolonged circulation time in blood and enhanced uptake by tumors.. Proc Natl Acad Sci U S A.

[OCR_01059] Gabizon A., Shiota R., Papahadjopoulos D. (1989). Pharmacokinetics and tissue distribution of doxorubicin encapsulated in stable liposomes with long circulation times.. J Natl Cancer Inst.

[OCR_01075] Huang S. K., Mayhew E., Gilani S., Lasic D. D., Martin F. J., Papahadjopoulos D. (1992). Pharmacokinetics and therapeutics of sterically stabilized liposomes in mice bearing C-26 colon carcinoma.. Cancer Res.

[OCR_01080] Klibanov A. L., Maruyama K., Torchilin V. P., Huang L. (1990). Amphipathic polyethyleneglycols effectively prolong the circulation time of liposomes.. FEBS Lett.

[OCR_01090] Madden T. D., Harrigan P. R., Tai L. C., Bally M. B., Mayer L. D., Redelmeier T. E., Loughrey H. C., Tilcock C. P., Reinish L. W., Cullis P. R. (1990). The accumulation of drugs within large unilamellar vesicles exhibiting a proton gradient: a survey.. Chem Phys Lipids.

[OCR_01085] Madden T. D., Tilcock C. P., Wong K., Cullis P. R. (1988). Spontaneous vesiculation of large multilamellar vesicles composed of saturated phosphatidylcholine and phosphatidylglycerol mixtures.. Biochemistry.

[OCR_01107] Mayer L. D., Bally M. B., Cullis P. R. (1986). Uptake of adriamycin into large unilamellar vesicles in response to a pH gradient.. Biochim Biophys Acta.

[OCR_01123] Mayer L. D., Bally M. B., Cullis P. R., Wilson S. L., Emerman J. T. (1990). Comparison of free and liposome encapsulated doxorubicin tumor drug uptake and antitumor efficacy in the SC115 murine mammary tumor.. Cancer Lett.

[OCR_01112] Mayer L. D., Tai L. C., Ko D. S., Masin D., Ginsberg R. S., Cullis P. R., Bally M. B. (1989). Influence of vesicle size, lipid composition, and drug-to-lipid ratio on the biological activity of liposomal doxorubicin in mice.. Cancer Res.

[OCR_01135] Morgan J. R., Williams L. A., Howard C. B. (1985). Technetium-labelled liposome imaging for deep-seated infection.. Br J Radiol.

[OCR_01146] Ogihara I., Kojima S., Jay M. (1986). Differential uptake of gallium-67-labeled liposomes between tumors and inflammatory lesions in rats.. J Nucl Med.

[OCR_01151] Olson F., Mayhew E., Maslow D., Rustum Y., Szoka F. (1982). Characterization, toxicity and therapeutic efficacy of adriamycin encapsulated in liposomes.. Eur J Cancer Clin Oncol.

[OCR_01162] Pestalozzi B., Schwendener R., Sauter C. (1992). Phase I/II study of liposome-complexed mitoxantrone in patients with advanced breast cancer.. Ann Oncol.

[OCR_01167] Pick U. (1981). Liposomes with a large trapping capacity prepared by freezing and thawing of sonicated phospholipid mixtures.. Arch Biochem Biophys.

[OCR_01177] Rahman A., Kessler A., More N., Sikic B., Rowden G., Woolley P., Schein P. S. (1980). Liposomal protection of adriamycin-induced cardiotoxicity in mice.. Cancer Res.

[OCR_01182] Schwendener R. A., Fiebig H. H., Berger M. R., Berger D. P. (1991). Evaluation of incorporation characteristics of mitoxantrone into unilamellar liposomes and analysis of their pharmacokinetic properties, acute toxicity, and antitumor efficacy.. Cancer Chemother Pharmacol.

[OCR_01193] Shenkenberg T. D., Von Hoff D. D. (1986). Mitoxantrone: a new anticancer drug with significant clinical activity.. Ann Intern Med.

[OCR_01206] Williams B. D., O'Sullivan M. M., Saggu G. S., Williams K. E., Williams L. A., Morgan J. R. (1986). Imaging in rheumatoid arthritis using liposomes labelled with technetium.. Br Med J (Clin Res Ed).

